# Schwannoma of the stomach: a case report

**DOI:** 10.1186/s13256-015-0788-0

**Published:** 2016-01-15

**Authors:** Aminder Singh, Ankur Mittal, Bhavna Garg, Neena Sood

**Affiliations:** Department of Pathology, Dayanand Medical College & Hospital, Tagore Nagar, Ludhiana, Punjab 141001 India

**Keywords:** GIST, Immunohistochemistry, Schwannoma, Stomach

## Abstract

**Background:**

Schwannomas, also known as neurilemmomas, are benign slow-growing neoplasms originating from a Schwann cell sheath. These neoplasms are rare among the mesenchymal tumors of the gastrointestinal tract. In the stomach, Schwannomas only represent 0.2 % of all gastric tumors; this makes the presentation of a schwannoma in the stomach of a man in his seventh decade unusual. This case report highlights the rarity of a schwannoma at the greater curvature of the stomach because only a few cases have been reported in the literature. This case describes the importance of including gastric schwannomas in the differential diagnosis when preoperative assessment reveals a submucosal gastric mass with gastrointestinal stromal tumor as a leading differential diagnosis because of its common occurrence at this site.

**Case presentation:**

A 72-year-old man of Indian origin presented with a painless abdominal mass with nonspecific gastrointestinal upset. An endoscopy showed a submucosal lesion in his stomach measuring 4×3×2 cm. Histology revealed a benign spindle cell tumor. Immunohistochemistry confirmed the diagnosis of gastric schwannoma. He is on regular follow-up and doing well.

**Conclusions:**

It should be remembered that a schwannoma can present as a mass lesion in the stomach and mimic gastrointestinal stromal tumor. Patients should undergo an endoscopy and a biopsy of the lesion should be done. Many patients do not undergo endoscopy which can delay diagnosis and management. As these tumors have an excellent prognosis, surgical removal is sufficient treatment. Surgeons, radiologists, pathologists and gastroenterologists must be aware of this entity.

## Background

Schwannomas, also known as neurilemmomas, are benign slow-growing neoplasms originating from a Schwann cell sheath. These neoplasms are rare among the mesenchymal tumors of the gastrointestinal tract but develop most commonly in the stomach representing 0.2 % of all gastric tumors [1–6]. The main differential diagnosis of a gastric submucosal schwannoma is a gastrointestinal stromal tumor (GIST). However, treatment and prognosis for gastric schwannomas and GISTs vary because gastric schwannomas have an excellent prognosis because of their benign nature whereas 10 to 30 % of GISTs can have malignant behavior with common recurrences. Malignant schwannomas of the stomach are extremely rare. Hence, it is important to make an accurate diagnosis for proper treatment options as medical management is available in the form of imatinib for GIST as compared to schwannoma, as well as surgical intervention in both [6, 7]. Patients can present with bleeding or a palpable mass. Schwannomas present as submucosal tumors and diagnosis is based on histological features and positivity for S-100 and negativity for smooth muscle actin (SMA) and c-Kit [3, 6–8]. As these tumors have an excellent prognosis, surgical removal is sufficient treatment.

## Case presentation

**Fig. 1:**
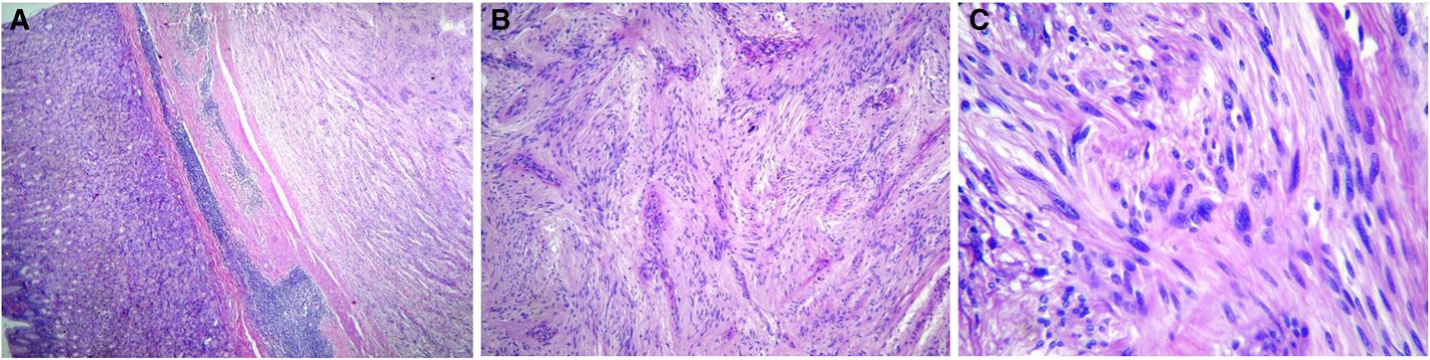
**a** Tumor present in the submucosal region of the stomach (hematoxylin and eosin ×100). **b** Interlacing bundles of spindle cells had elongated palisading nuclei with ill-defined cytoplasmic borders (hematoxylin and eosin ×200). **c** Close-up showing mild nuclear pleomorphism with spindled-shaped nuclei and hypocellular regions (hematoxylin and eosin ×400)

**Fig. 2:**
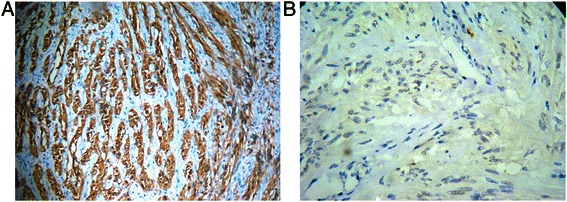
**a** Immunohistochemistry showed strong S-100 positivity. **b** Immunohistochemistry showed c-Kit negativity

We present the case of a 72-year-old man of Indian origin who came to our hospital with complaints of a painless mass with abdominal fullness. There was no significant past medical, family, or psychosocial history. There was no history of upper gastrointestinal bleed, or past medical or surgical interventions. On physical examination, he had mild pallor with ill-defined painless swelling in his gastric region. There was no organomegaly or lymphadenopathy. An upper gastrointestinal endoscopy was done which was suggestive of a submucosal lesion in his stomach. Our gastroenterologist thought of GIST; leiomyoma and even gastric malignancy was also suspected because of the patient’s old age. A subsequent biopsy was taken which revealed only mucosal tissue. A computed tomography (CT) scan of the patient’s abdomen showed an ovoid homogenous 4×3×2 cm mass at the greater curvature of his stomach. There was no other lesion in his whole body. We finally suspected submucosal tumor with the possibility of GIST. Considering the large size of the tumor, location, difficulty of establishing a definite preoperative diagnosis in spite of incision biopsy, suspicion of malignant GIST/tumor, old age and to prevent possible complications such as bleeding or pyloric stenosis, surgical intervention was planned and a subtotal gastrectomy was done. The surgical specimen was received in the department of pathology. On gross examination, an intramural, nodular, solid mass measuring 4×3×2 cm was seen. A cut section revealed whorling trabeculation. Histological observations showed a cellular tumor present in the submucosa, which extended into his muscularis propria. The overlying mucosa was unremarkable. Sections from the tumor showed interlacing bundles of spindle cells which had elongated nuclei, ill-defined cytoplasmic borders and palisading nuclei. Mild nuclear atypia was noted. These cells were separated by collagenous strands. Mitotic activity 5/50 high-power fields were seen; however, no necrosis/atypical mitosis were identified (Fig. 1). There was no lymph node involvement and the surgical margin was negative for tumor cells. A histological diagnosis of a benign mesenchymal tumor was made. Immunohistochemistry (IHC) staining was strongly positive for vimentin and S-100 (Fig. 2a), whereas c-Kit (Fig. 2b), SMA and cytokeratin (CK) were negative. Hence, a final diagnosis of schwannoma was made. His postoperative period was uneventful and he was discharged in good condition. Follow-up visits up to 1.5 years were unremarkable.

## Discussion

The vast majority of spindle cell stromal tumors of the gastrointestinal tract were once considered to originate from smooth muscle. With the development of immunohistochemical staining and electron microscopy various distinct origins have become apparent. These have allowed for a more precise classification among stromal tumors of the gastrointestinal tract. The most common gastrointestinal site is the stomach. Schwannomas constitute 0.2 % of all gastric neoplasms [1]. Malignant transformation of a gastric schwannoma is very rare. These tumors arise from the fundus, body or antrum of the stomach and are commonly intramural. Tumors can vary from 0.5 to 11 cm in diameter. Schwannomas tend to present in the fifth to sixth decades of life and more commonly in females. They are usually asymptomatic and can be discovered incidentally. They are usually solitary lesions arising from the lesser curvature of the stomach [2–4]; however, in our case it was the greater curvature. The most common presenting symptom can be upper gastrointestinal bleeding and abdominal discomfort. On a CT scan, a gastric schwannoma tends to be homogenous, which distinguishes it from leiomyomas and leiomyosarcomas. Magnetic resonance imaging (MRI) can determine the exact layer of origin and location of the tumor [8]. The typical endoscopic appearance of gastric schwannoma is a round protruding submucosal mass with overlying ulcerated mucosa which is usually seen in patients with a history of gastrointestinal bleeding. In our case, an initial biopsy revealed only mucosal tissue; false-negative results of an endoscopic biopsy can be given because normal mucosa overlies the submucosal lesion. On pathological examination the tumors are encased by intact mucosa and they principally involve submucosa and muscularis propria. These tumors have spindle-shaped nuclei and have a fascicular arrangement. No mitosis/necrosis/significant nuclear pleomorphism are seen. A diffuse and intense positivity for vimentin and S-100 protein is detected. Gastric malignant schwannomas can be distinguished from benign schwannomas on the basis of histological examination of the resected specimens, not by clinical symptoms or imaging studies [3, 9–11]. The treatment options for gastric schwannoma are the same regardless of whether the tumor is benign or has malignant potential. Thus, surgical resection is the only possible treatment for gastric schwannoma as compared to GIST where larger tumors necessitate subtotal or total gastrectomy [12, 13].

## Conclusions

It should be remembered that a schwannoma can present as a mass lesion in the stomach and can mimic GIST. Many patients do not undergo endoscopy which can delay diagnosis and management. As these tumors have an excellent prognosis, surgical removal in the form of wedge resection, subtotal resection or near-total resection is sufficient treatment. The prognosis for patients with a solitary schwannoma of the stomach following resection is very good. Surgeons, radiologists, pathologists and gastroenterologists must be aware that although gastric schwannomas are rare among spindle cell tumors of the gastrointestinal tract, diagnosis should always be confirmed by IHC and GISTs should be ruled out. Although malignant transformation can occur, it is very rare. Recurrent disease is generally associated with an incomplete surgical margin. The use of molecular therapy for gastric schwannoma is not clearly established yet.

## Consent

Written informed consent was obtained from the patient for publication of this case report and accompanying images. A copy of the written consent is available for review by the Editor-in-Chief of this journal.

## References

[1] Miettinen M, Majidi M, Lasota J. Pathology and diagnostic criteria of gastrointestinal stromal tumors (GISTs): a review. Eur J Cancer. 2002;38 Suppl 5:39–51. 10.1016/s0959-8049(02)80602-5 12528772

[2] Sarlomo-Rikala M, Miettinen M. Gastric schwannoma – a clinicopathological analysis of six cases. Histopathology. 1995;27(4):355–60. 10.1111/j.1365-2559.1995.tb01526.x 8847066

[3] Fletcher CD, Berman JJ, Corless C, Gorstein F, Lasota J, Longley BJ, et al. Diagnosis of gastrointestinal stromal tumors: a consensus approach. Hum Pathol. 2002;33(5):459–65. 10.1053/hupa.2002.123545 12094370

[4] Ueyama T, Guo KJ, Hashimoto H, Daimaru Y, Enjoji M. A clinicopathological and immunohistochemical study of gastrointestinal stromal tumors. Cancer. 1992;69(4):947–55. 10.1002/1097-0142(19920215)69:4<947::aid-cncr2820690419>3.0.co;2-x 1735086

[5] Lin C-S, Hsu H-S, Tsai C-H, Li W-Y, Huang M-H. Gastric schwannoma case report. J Chin Med Assoc. 2004;67:583–6. 15720074

[6] Prévot S, Bienvenu L, Vaillant JC, de Saint-Maur PP. Benign schwannoma of the digestive tract: a clinicopathologic and immunohistochemical study of five cases, including a case of esophageal tumor. Am J Surg Pathol. 1999;23(4):431–6. 10.1097/00000478-199904000-00007 10199472

[7] Yoon W, Paulson K, Mazzara P, Nagori S, Barawi M, Berri R. Gastric schwannoma: a rare but important differential diagnosis of a gastric submucosal mass. Case Rep Surg. 2012;2012:280982. 10.1155/2012/280982 PMC3423772 22924149

[8] Melvin WS, Wilkinson MG. Gastric schwannoma – clinical and pathologic considerations. Am Surg. 1993;59(5):293–6. 8489097

[9] Miettinen M. Gastrointestinal stromal tumors. An immunohistochemical study of cellular differentiation. Am J Clin Pathol. 1988;89(5):601–10. 10.1093/ajcp/89.5.601 3282426

[10] Sakai F, Sone S, Yanagisawa S, Ishii Z. Schwannoma of lesser omentum. Eur J Radiol. 1988;8(2):113–4. 3289925

[11] Muhammad A. An unusual case of gastric schwannoma. J Coll Phys Surg Pak. 1997;7(2):80–1.

[12] Atmatzidis S, Chatzimavroudis G, Dragoumis D, Tsiaousis P, Patsas A, Atmatzidis K. Gastric schwannoma: a case report and literature review. Hippokratia. 2012;16(3):280–2. PMC3738740 23935300

[13] Kan Y-K, Kang HJ, Kim K-M, Sohn T, Choi D, Ryu M-H, et al. Clinical practice guideline for accurate diagnosis and effective treatment of gastrointestinal stromal tumor in Korea. Cancer Res Treat. 2012;44(2):85–96. 10.4143/crt.2012.44.2.85 PMC3394868 22802746

